# How (Not) to Sleepwalk into the Next Pandemic!

**DOI:** 10.1007/s40319-023-01332-9

**Published:** 2023-05-30

**Authors:** Felix Addor

**Affiliations:** Deputy Director General of the Swiss Federal Institute of Intellectual Property and Professor of International Negotiations, University of Bern, Bern, Switzerland

Thanks to an unprecedented innovation effort in virology and immunology, COVID-19 vaccines, therapeutics and diagnostics were rapidly brought to the market in less than 12 months after the pandemic began.^1^ Even though many have associated the research and development of the new vaccines, therapeutics and diagnostics with a handful of companies, in reality hundreds of development and manufacturing partnerships quickly formed around the globe. Today, hundreds of COVID-19 vaccines candidates are in preclinical and clinical development.^2^

Admittedly, there was a shortage of vaccines at the start of the COVID-19 pandemic, and access was not guaranteed in the same way for all countries. This is why, back in October 2020, at a time when no COVID-19 vaccine was yet available, a group of more than 60 member states of the World Trade Organization (WTO) led by South Africa and India called for the suspension of the WTO Agreement on Trade-Related Aspects of Intellectual Property Rights (TRIPS Agreement).^3^ They complained that international intellectual property protection hindered their access to innovative medical products. They requested a so-called “TRIPS waiver”, arguing that it would exempt them from patent restrictions and allow each country itself to manufacture these products, thus ensuring access without restrictions imposed by international law.

The WTO discussed the demand for this so-called TRIPS waiver intensively and controversially for 20 months. At the 12th Ministerial Conference (MC12) in Geneva in June 2022, member states reached an agreement.^4^ While it provides for (additional) simplifications in the granting of compulsory licenses on COVID-19 vaccines for developing countries, there was no consensus among the WTO members for a TRIPS waiver as initially requested.

For the international IP system, this was a positive outcome, a kind of “damage control”. As the Max Planck Institute for Innovation and Competition has convincingly argued, waiving IP rights would not have accelerated the production and distribution of the vaccines.^5^ Rather, the lack of access to COVID-19 vaccines was due to practical problems. The establishment and expansion of the necessary production capacities and the international supply chains to procure the limited raw materials available, as well as the formation of the production partnerships, were carried out in record time. Now, in spring 2023, the capacity for COVID-19 vaccines is sufficient to vaccinate every person on earth five times over – every year.^6^

This would not have happened without clear international rules on patent protection. The existing strong IP system contributed to the containing of the COVID-19 pandemic in two ways. Firstly, it fulfilled its classic role of providing incentives for companies to make major investments. Pharmaceutical companies knew that if they could successfully develop and market a product, they would be able to sell large quantities and recoup their huge upfront investments. In an interview, Patrick Tippoo, Executive Director of the African Vaccine Manufacturing Initiative and Head of Science and Innovation of South Africa’s biopharmaceutical company Biovac, warned of what would happen if the IP regime was weakened: “Without IP, such investment will dry up. I don’t see how it would be possible to incentivize investors, manufacturers and developers to risk their time, effort and money without protecting the reward that comes from that.”^7^ Secondly, IP has enabled companies that would otherwise compete to collaborate on R&D and thereby to jointly contribute to the containment of the pandemic. For example, without international IP rules, Lonza would not have produced the active ingredient for Moderna’s vaccine, Pfizer and BioNTech would not have collaborated either, and Oxford University and Astra Zeneca would not have entered into manufacturing partnerships and supply contracts through licensing agreements with generic companies in India and other regions of the world. In short, recent experience shows that COVID-19 vaccines were developed not *in spite of* IP, but rather *because of* it.

At this very moment, WTO members are discussing whether to extend the MC12 TRIPS decision to include therapeutic and diagnostic products. It seems that we are facing a déjà vu at the TRIPS Council: as with the original TRIPS waiver proposal, we see a positional exchange of arguments that are hard to reconcile. Consensus seems elusive. It is highly unfortunate that the lessons that could and should have been learned from the COVID-19 pandemic are being ignored not only in WTO conference rooms but also in other fora such as the Intergovernmental Negotiating Body for an international instrument on pandemic prevention, preparedness and response at the WHO. In addition, large shares of civil society seem to ignore the developments that have taken place since the outbreak of the pandemic. For example, in October 2022, a group of illustrious persons signed “The People’s Vaccine”^8^ letter in which they, among other things, call for a waiver of IP provisions because this “would dramatically improve access to all these lifesaving products.”

It is not only in the context of the COVID-19 pandemic that waiving IP rights is considered a panacea for all problems. This pertains to international cooperation in the broader sense. However, it would amount to throwing out the baby with the bathwater. Suspending an international legal framework, which has been in force for nearly 30 years and accepted by 164 states during a global pandemic would send a problematic signal to the international community. How will countries be able to effectively address global challenges, such as climate change, poverty reduction or migration if the international rules accepted by all ultimately risk to be waived in the event of a crisis? This would destroy the reliability and predictability created by the international legal order. That this scenario is not a pipe dream was exemplified, among other things, by the remarks of WTO Director General Okonjo-Iweala on 7 February 2023 in a talk at the London School of Economics. When asked whether she would “support an intellectual property waiver for climate technologies in order to tackle the climate crisis”, she answered that “on the green tech, I could not agree more. We’re going to see more of this type of argument.”^9^ And this “more” is just around the corner: currently, TRIPS Council members are discussing whether to adopt a “trigger mechanism” that would *automatically* waive IP provisions for future pandemics or other public health emergencies. The latter term is sufficiently vague so that each and every situation could be subsumed under it.

Even though COVID-19 is broadly viewed as being a “once in a lifetime” or “once in a century” pandemic, some fear that the next pandemic could come much sooner and be more severe than we think.^10^ This underlines the urgency that instead of wasting further time, the international community should draw its conclusions from the COVID-19 pandemic and focus on *what really matters: preparing for future public health or other emergencies*. A thorough and honest analysis is needed, without any ideological bias or ulterior motives. Elements of such a discussion should include the following ideas:

- The WTO needs to establish clear rules to secure supply chains and reduce export restrictions. For example, they have severely hampered access to COVID-19 vaccines for developing countries and Africa in particular, including forcing the international COVAX program to reduce its 2021 delivery target from two billion to 930 million doses.^11^ This was because the generic company Serum Institute of India, which COVAX had contracted to manufacture COVID-19 vaccines under license from Astra Zeneca, could no longer supply them due to national export restrictions.^12^
- The WTO should create a workable mechanism to ensure the equitable distribution of vaccines and other medical products in the event of a future pandemic, so that the Global South receives them in a timely manner. For its part, the biopharmaceutical industry has committed to this goal in its “Berlin Declaration”.^13^
- The production of vaccines, therapeutics and other medical products should be further diversified geographically. Some companies have already taken steps in this direction. BioNTech, for example, began construction of its African mRNA vaccine manufacturing hub in Rwanda in June 2022.^14^ Steps like these will ensure a better regional supply of vaccines and other medical products in the event of a pandemic, especially in sub-Saharan Africa.
- The WTO, WHO and WIPO should establish a matchmaking platform to facilitate licensing agreements and close collaboration between innovators and qualified manufacturers, thereby contributing actively and more effectively to a global manufacturing scale-up than a mere technology pool. It is important to understand the specific technical ecosystems in each region and country, so that production capacity can be expanded quickly without the need to search for potential partners.
- States need to ensure early and free access to gene sequences in case of outbreaks.
- The WHO should significantly increase its support to countries to improve their health infrastructure. The COVID-19 pandemic revealed major deficiencies in the distribution of medical products, especially outside major cities.
- Regulatory processes should be streamlined. In an emergency situation, medical products should be able to be approved for a larger region by the regulatory authority of a specific, pre-determined country. This would allow earlier market entry in all countries in the region.
- Governments should condition all public R&D funding and licensing of publicly-funded innovations as dependent on conditions relating to access, affordability and technology transfer, rather than trying to fix these problems in hindsight.

All this can only be achieved in a sustainable manner in close partnership with innovators and on the basis of international law. Disrupting the international system of IP protection will not advance the goal of pandemic preparedness. Quite on the contrary! By destroying predictability and confidence, the whole world, including the Global South, would be worse off.

It is up to us to avoid sleepwalking into situations for which we would be even less prepared than with the COVID-19 pandemic, and for which no rapid improvement can be expected.

## Footnotes

1 The genetic sequence of the SARS-CoV-2 virus was published on 11 January 2020. Pfizer/BioNTech were the first companies to receive a market authorization for their vaccine on 2 December 2020.

2 According to the WHO COVID-19 vaccine tracker and landscape, 183 COVID-19 vaccines candidates are in clinical and 199 in pre-clinical development; https://www.who.int/publications/m/item/draft-landscape-of-covid-19-candidate-vaccines, last accessed on 14 April 2023.

3 WTO TRIPS Council Document IP/C/W/669 of 02.10.2020, revised by WTO TRIPS Council Document IP/C/W/669/Rev.1 of 25 May 2021.

4 Ministerial Declaration on the WTO response to the COVID-19 pandemic and preparedness for the future, WTO Document WT/MIN(22)/31, WT/L/1142 of 22 June 2022.

5 Reto M. Hilty, Pedro Henrique D. Batista, Suelen Carls, Daria Kim, Matthias Lamping and Peter R. Slowinski, “COVID-19 and the Role of Intellectual Property: Position Statement of the Max Planck Institute for Innovation and Competition of 7 May 2021”, Max Planck Institute for Innovation & Competition Research Paper No. 21-13, available at SSRN: https://ssrn.com/abstract=3841549 or http://dx.doi.org/10.2139/ssrn.3841549.

6 UNICEF COVID-19 Market Dashboard, https://www.unicef.org/supply/covid-19-market-dashboard, last accessed on 13 April 2023.

7 “Why vaccine independence is so important for Africa”, WIPO Magazine, December 2022, https://www.wipo.int/wipo_magazine/en/2022/04/article_0005.html, last accessed on 14 April 2023.

8 THE PEOPLE’S VACCINE Never Again, https://peoplesvaccine.org/wp-content/uploads/2023/03/Peoples-Vaccine-Alliance-3-years-Never-Again-8.pdf, last accessed 14 April 2023.

9 Marc L Busch, “Seventeen seconds that could spell the end of international intellectual property rights”, The Hill, 11 March 2023, https://thehill.com/opinion/international/3894445-seventeen-seconds-that-could-spell-the-end-of-international-intellectual-property-rights/, last accessed on 14 April 2023.

10 Eleni Smitham and Amanda Glassman, “The Next Pandemic Could Come Soon and Be Deadlier”, Center for Global Development Blog Post, https://www.cgdev.org/blog/the-next-pandemic-could-come-soon-and-be-deadlier, last accessed on 14 April 2023.

11 “COVAX Delivers One Billionth COVID-19 Vaccine Dose – But 41% of World Remains Unvaccinated”, Health Policy Watch, https://healthpolicy-watch.news/covax-delivers-one-billionth-covid-19-vaccine-but/, last accessed on 14 April 2023.

12 Financial Times, “India blocks vaccine exports in blow to dozens of nations”, 25 March 2021, https://www.ft.com/content/5349389c-8313-41e0-9a67-58274e24a019, last accessed on 14 April 2023.

13 https://ifpma.org/wp-content/uploads/2023/01/i2023_IFPMA_Berlin-Declaration_Biopharmaceutical-industry-vision-for-equitable-access-in-pandemics-1.pdf, last accessed on 14 April 2023.

14 “BioNTech launches African vaccine production in Rwanda”, https://www.europeanpharmaceuticalreview.com/news/172615/biontech-launches-african-vaccine-production-in-rwanda/, last accessed on 14 April 2023.

